# Rapid generation advancement in soybean: approaches, constraints, and the stage-dependent photoperiod window

**DOI:** 10.3389/fpls.2026.1992171

**Published:** 2026-10-30

**Authors:** Yawar Habib, Altafur Rahman, Gabriele Adornato, Elshan Musazade, Anshika Bhatla, Rachna Gowlikar, Avanish Rai, Tsanko Gechev, Xianzhong Feng, Elena Potokina

**Affiliations:** 1 Center for Bio and Medical Technologies, Skolkovo Institute of Science and Technology, Moscow, Russia; 2 Center of Plant Systems Biology and Biotechnology, Plovdiv, Bulgaria; 3 Key Laboratory of Soybean Molecular Design Breeding, Northeast Institute of Geography and Agroecology, Chinese Academy of Sciences, Changchun, China; 4 Institute of Molecular Plant Sciences, University of Edinburgh, Scotland, United Kingdom; 5 School of Biology and Environmental Science and UCD Earth Institute, University College Dublin, Dublin, Ireland; 6 Department of Molecular Biology, University of Plovdiv, Plovdiv, Bulgaria; 7 Institute of Forest and Natural Resources Management, Saint Petersburg State Forest Technical University, St. Petersburg, Russia

**Keywords:** soybean, speed breeding, rapid generation advancement, photoperiod, *E1*, flowering time, genomic selection, controlled environment

## Abstract

Long generation intervals constrain realized genetic gain in soybean (*Glycine max* (L.) Merr.) breeding. Speed-breeding protocols developed for long-day and day-neutral crops use an extended photoperiod throughout the entire life cycle. They are not directly applicable to soybean, because the *E1* family represses the florigen genes *GmFT2a* and *GmFT5a* under long days and delays flowering. Rapid generation advancement in soybean therefore depends on a stage-dependent photoperiod window. Before flowering, the photoperiod (10–12 h in published stage-dependent protocols) must remain below the genotype-specific critical photoperiod, and an extended photoperiod can be introduced only after flowering (R1 stage), to accelerate seed maturation. Optimized continuous short-day systems are reported to allow up to five generations per year, and six are reported by one study that combined a post-flowering 22-h photoperiod with germination of immature seed. These figures come from different studies, genotypes and facilities. To our knowledge, no study has yet compared cycle length under a post-flowering long day with a corresponding short-day control in the same genotypes. A post-flowering 22-h photoperiod also reduced leaf photosynthetic capacity, and applying the same early harvest date to all plants may change allele frequencies in segregating populations. This review summarizes soybean speed-breeding protocols, their infrastructure requirements, and their physiological and genetic limitations. It also evaluates the integration of these protocols with marker-assisted selection, genomic selection and genome editing. Early seed harvest and high-density planting can be transferred between crops, but the photoperiod regime must be tailored to soybean. We conclude that the evidence supports soybean speed breeding as a method for accelerating generation turnover, and that phenotypic selection in the chamber needs to be validated in the field. Published controlled-environment soybean protocols do not report changes in allele frequency during accelerated advancement or genetic correlations between traits measured in the chamber and in the field. Both are needed to show whether rapid cycling translates into genetic gain in the field.

## Graphical Abstract

**Figure:**
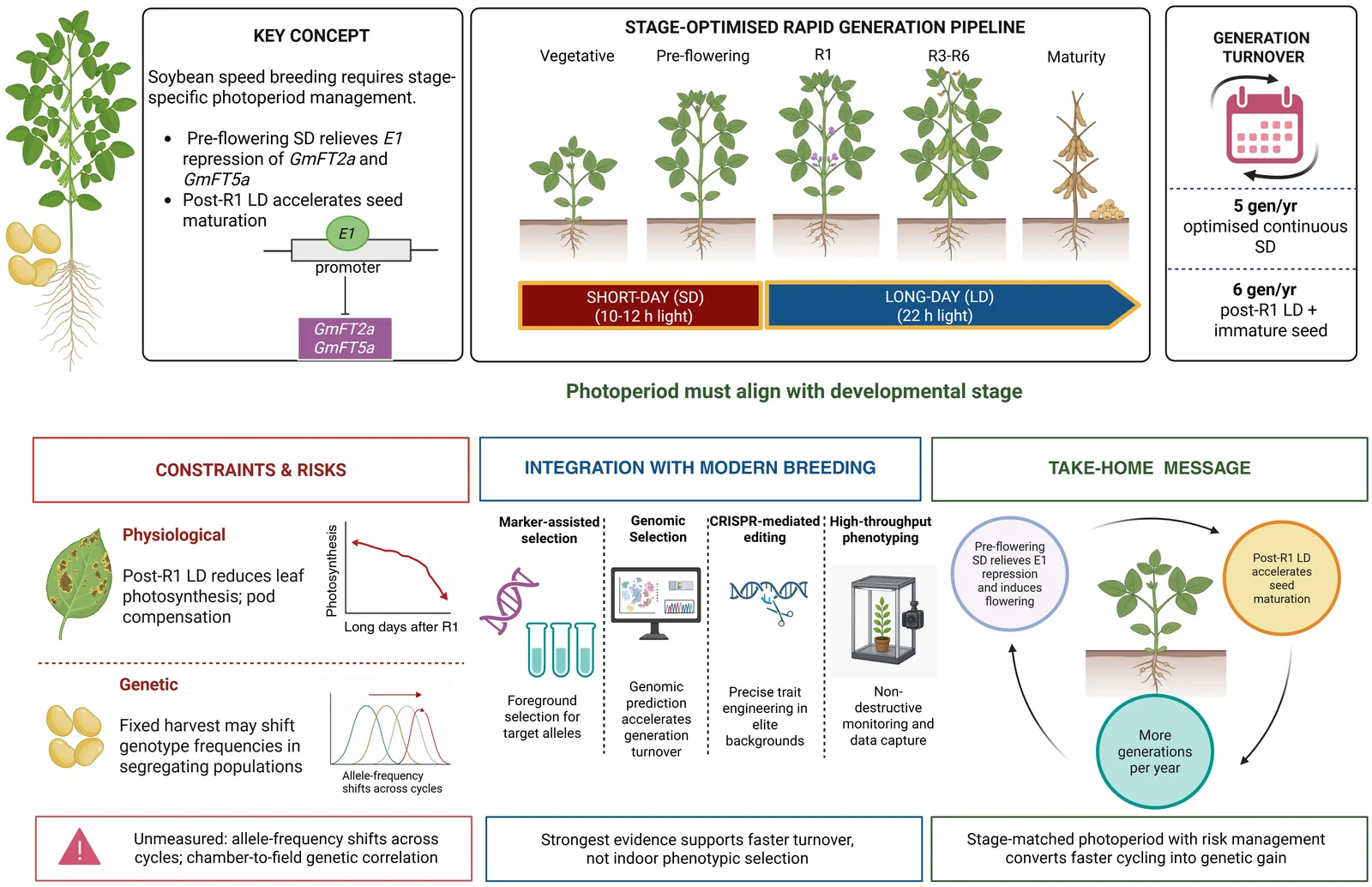

## 1 Introduction

Soybean (*Glycine max* (L.) Merr.) is the most widely produced oilseed crop worldwide and a major source of plant-based protein. Of the 429.5 million tonnes of soybeans produced globally in 2025/26, approximately 80% were produced in Brazil, the United States, and Argentina (United States Department of Agriculture, 2026). However, average global farm yields remain at 2770–2790 kg ha^-^¹, with yield stagnation across 23% of the harvested area (Nawaz et al., 2026). Yield gaps leave 44% of potential production unrealized in key regions such as southern Brazil, while global caloric requirements are projected to increase by 46.8% by 2050 (Nawaz et al., 2026). Addressing these challenges requires the faster development of higher-yielding and more stress-tolerant cultivars than is currently possible through conventional breeding.

Generation time remains a major constraint in cultivar development, as breeding lines must progress through successive generations. Following a cross between two homozygous lines, six generations of self-pollination are required to achieve 98.4% homozygosity, taking approximately three years at two generations per year (Ghosh et al., 2018). This limitation is particularly relevant to soybean, as long-duration varieties may require approximately 120 days to reach maturity (Rajendran et al., 2025), while field and greenhouse conditions generally permit only one or two generations per year (Nagatoshi and Fujita, 2019). According to the breeder’s equation, annual genetic gain is inversely related to breeding cycle length, highlighting the importance of reducing generation time (Aggarwal et al., 2025).

One approach to shortening breeding cycles is rapid generation advancement (RGA), or speed breeding. Protocols based on extended photoperiods have been particularly successful in long-day and day-neutral crops. Watson et al. (2018) reported that speed breeding can achieve up to six generations per year in spring wheat, durum wheat, barley, chickpea, and pea, and four in canola, compared with two to three under normal glasshouse conditions. Harvesting and germinating immature seeds can further shorten generation intervals without embryo rescue or plant growth regulators (Ghosh et al., 2018). Speed breeding can improve selection accuracy and accelerate genetic gain, but it requires advanced infrastructure, substantial investment, and skilled personnel (Kayess et al., 2025). These requirements limit its adoption, particularly by smaller breeding enterprises and in resource-limited countries.

Extended-photoperiod protocols cannot be applied directly to soybean, because its flowering is highly sensitive to photoperiod and regulated by a complex network of maturity-related genes. However, successful soybean RGA depends on more than short photoperiods alone. Nagatoshi and Fujita (2019) showed that appropriate light and temperature cycles accelerated flowering in a CO_2_-supplemented growth chamber, while CO_2_ enrichment enhanced plant growth and yield. Jähne et al. (2020) developed an LED-based protocol combining short photoperiods with an adjusted light spectrum, producing short, sturdy plants suitable for multi-tier systems. Although far-red light accelerated flowering in some amaranth and rice genotypes, it had no effect on soybean flowering and caused undesirable petiole elongation, supporting its exclusion from soybean protocols (Jähne et al., 2020).

The timing of photoperiod manipulation is another important consideration. The highest reported number of generations in soybean comes from a two-stage protocol in which day length is extended after flowering begins. Aciksoz et al. (2025) maintained plants under a 12-h photoperiod until the R1 stage (approximately 24 days after sowing), and then extended the photoperiod to 22 h. Short days promote floral induction by alleviating *E1*-mediated repression of *FT* florigen genes (Xia et al., 2012). However, photoperiod effects extend beyond floral initiation, as longer days during the reproductive phase can also influence subsequent developmental processes, particularly pod formation (Sun et al., 2024).

Responses to photoperiod manipulation may also vary with maturity alleles, which have been extensively studied in soybean adapted to high latitudes. In Russia, a short frost-free period makes early maturity the principal breeding priority in the Far East (Habib et al., 2026), while long summer photoperiods limit expansion into Siberia, where photoperiod-insensitive cultivars are required (Perfil’ev et al., 2024). In northern Kazakhstan (53° N), marker-assisted selection for recessive maturity alleles produced early-maturing breeding lines that matured within 92–102 days, all of which carried recessive alleles at *E1* and *E7* (Yerzhebayeva et al., 2023). Among 438 high-latitude accessions evaluated at and beyond 50° N in Northeast China, all carried the *e1-as* allele of *E1*, and functional mutations were also identified in *E2*, *E3* (*GmPHYA3*) and *E4* (*GmPHYA2*) (Jia et al., 2025). For Central European breeding material, a marker array targeting *E1–E4*, *E7*, *E9*, and *E10* has been developed to support selection at these loci (Rychel-Bielska et al., 2025). Such genotypic differences are relevant to RGA because cultivars adapted to different latitudes may respond differently under identical growth-chamber conditions (Zhu et al., 2023).

Despite advances in marker-assisted selection (MAS) and the characterization of maturity loci, soybean-specific reviews of RGA remain limited. Recent speed breeding reviews have primarily adopted a multi-crop perspective (Aggarwal et al., 2025; Blinkov et al., 2025; Mladenov et al., 2026), and many established protocols were originally developed for long-day cereals. Jähne et al. (2020) included soybean in a broader assessment of short-day crops, alongside rice and amaranth. Furthermore, relatively few studies have directly compared soybean RGA protocols, and the protocols differ in how they report cycle length, generations per year and the field performance of advanced lines.

In this narrative review, we first examine the developmental stages at which photoperiod can be modified during soybean RGA, with particular attention to the *E1–E4* maturity loci and *FT* flowering pathway (Section 2). We then discuss RGA strategies and supporting technologies (Sections 3 and 4) and their physiological and developmental limitations (Section 5). We also discuss the integration of RGA with MAS, genomic selection and genome editing (Section 6), and compare soybean RGA protocols with those developed for other crops (Section 7). Where soybean data are lacking, we draw on findings from other crops and name the crop in each case.

## 2 Soybean photoperiod sensitivity and constraints on RGA

Soybean is a facultative short-day plant, and extending the photoperiod delays flowering. Kong et al. (2010) reported that flowering was markedly delayed under long days compared to short days. In the same study, two florigen genes that promote the transition to flowering, *GmFT2a* and *GmFT5a*, were strongly up-regulated under short days and peaked at 4 hours after dawn, whereas under long days they were down-regulated with no clear diurnal rhythm. The extended photoperiods used to accelerate wheat and barley may therefore have the opposite effect on soybean. The photoperiod response is genotype-dependent. Soybean germplasm is classified into 13 maturity groups, from MG 000 at high latitudes to MG X at low latitudes (Miladinović et al., 2018), and an earlier group, MG 0000, has since been proposed for very early germplasm from northern China and the Russian Far East (Yerzhebayeva et al., 2023). Allelic combinations at the four major maturity loci, *E1–E4*, contribute to this latitudinal adaptation (Miladinović et al., 2018).

*E1* has a major effect on flowering time. Positional cloning identified an intron-free gene encoding a protein with a putative bipartite nuclear localization signal and a region distantly related to the B3 domain. *E1* expression was suppressed under short days and bimodal under long days, peaking first about two hours after dawn (Xia et al., 2012; Zhai et al., 2015). A nonsynonymous substitution in the localization signal of the recessive allele impairs localization specificity and results in earlier flowering, and the same phenotype was observed in three ethylmethanesulfonate-induced mutants and two naturally occurring mutant alleles containing a premature stop codon or a deletion of the entire gene (Xia et al., 2012). *E1* transcript abundance was negatively correlated with that of *GmFT2a* and *GmFT5a*, and introduction of functional *E1* into the early-flowering cultivar Kariyutaka produced late flowering (Xia et al., 2012). Allelic variation at the locus was shown to underlie flowering time in three F2 populations (Zhai et al., 2015). The three *E1*-family genes arose through genome duplication and have since diverged in function: *E1* retains full repressive activity, whereas *E1La* and *E1Lb*, which differ in subcellular localization, are weaker repressors. Each also represses its own expression and that of the other two. *E1Lb* binds directly to the *GmFT2a* and *GmFT5a* promoters, as reported for *E1* and *E1La* (Fang et al., 2024).

The remaining three classical loci controlling maturity encode a *GIGANTEA* orthologue and two phytochromes. Map-based cloning identified *GmGIa* as the gene underlying *E2* (Watanabe et al., 2011). The recessive allele *e2* carries a premature stop codon in the 10th exon; a mutant line with a different premature stop codon flowered earlier than its original variety, Bay, and *e2/e2* plants showed elevated *GmFT2a* expression (Watanabe et al., 2011). *GmPHYA3* was identified as the gene responsible for *E3* (Watanabe et al., 2009). Long days with a high ratio of red to far-red light enhanced the effect of *E3* alleles across genetic backgrounds, and under such conditions, the mutant with a non-functional *GmPHYA3* gene flowered 15 days earlier than the wild-type Bay variety (Watanabe et al., 2009). The second phytochrome A gene, *GmPHYA2*, underlies *E4*, and the recessive *e4* allele, associated with photoperiod insensitivity, contains a *Ty1*/copia-type retrotransposon in the first exon (Liu et al., 2008). Its paralog *GmPHYA1* is located in a homeologous region, and *e4* homozygotes de-etiolate only partially under continuous far-red light, indicating that the mutation does not abolish phytochrome A function (Liu et al., 2008). A line with mutations in two *PHYTOCHROME A* genes produced high levels of *GmFT2a* and *GmFT5a* under long days and flowered slightly earlier under long days than the wild type under short days (Kong et al., 2010). The *E1*, *E3* and *E4* loci also respond differentially to light quality. In some genotypes, long days with a high ratio of red to far-red light did not delay flowering, and a line previously considered insensitive to long days was sensitive under a low ratio of red to far-red light (Cober et al., 1996).

The genes described above regulate members of the *FT* gene family. Soybean has ten *FT* homologues, arranged as five linked pairs (Kong et al., 2010). Unlike *GmFT2a* and *GmFT5a*, *GmFT4* is up-regulated under long days and down-regulated under short days. It is also induced in plants overexpressing *E1*, in which *GmFT2a* and *GmFT5a* are suppressed, and its overexpression delayed flowering in *Arabidopsis*, consistent with a role as a flowering repressor (Zhai et al., 2014). *GmFT2a* and *GmFT5a* are not functionally equivalent after floral induction: they induced flowering to a similar degree in ectopic overexpression assays, but *GmFT5a* was markedly more effective in terminating stem growth after flowering (Takeshima et al., 2019). Other maturity loci, including *E9* and *E10*, have been assigned to *FT* homologues (Rychel-Bielska et al., 2025).

The long-juvenile trait extends the vegetative phase and improves yield under short-day conditions, enabling cultivation in tropical regions (Lu et al., 2017). *J*, the major locus conferring this trait, was identified as the soybean orthologue of *Arabidopsis EARLY FLOWERING 3* (*ELF3*). *J* depends genetically on *E1*: the J protein physically associates with the *E1* promoter and down-regulates its transcription, which relieves the repression of *GmFT2a* and *GmFT5a* and promotes flowering under short days (Lu et al., 2017). Two pseudo-response regulator genes, *Tof11* and *Tof12*, act through *LHY* homologues to promote *E1* expression and delay flowering under long photoperiods, and *Tof12*-dependent acceleration of maturity during domestication may have facilitated latitudinal expansion (Lu et al., 2020). Photoperiod sensitivity is not confined to floral induction. Long days extended the time from flowering to pod formation, and moving plants from long to short days at the onset of flowering (R1) reversed the delay. Mutants lacking *E1*-family or Evening Complex genes showed no difference in pod formation time between photoperiods (Sun et al., 2024). The major photoperiod-responsive components are summarized in **Figure 1**.

**Figure 1:**
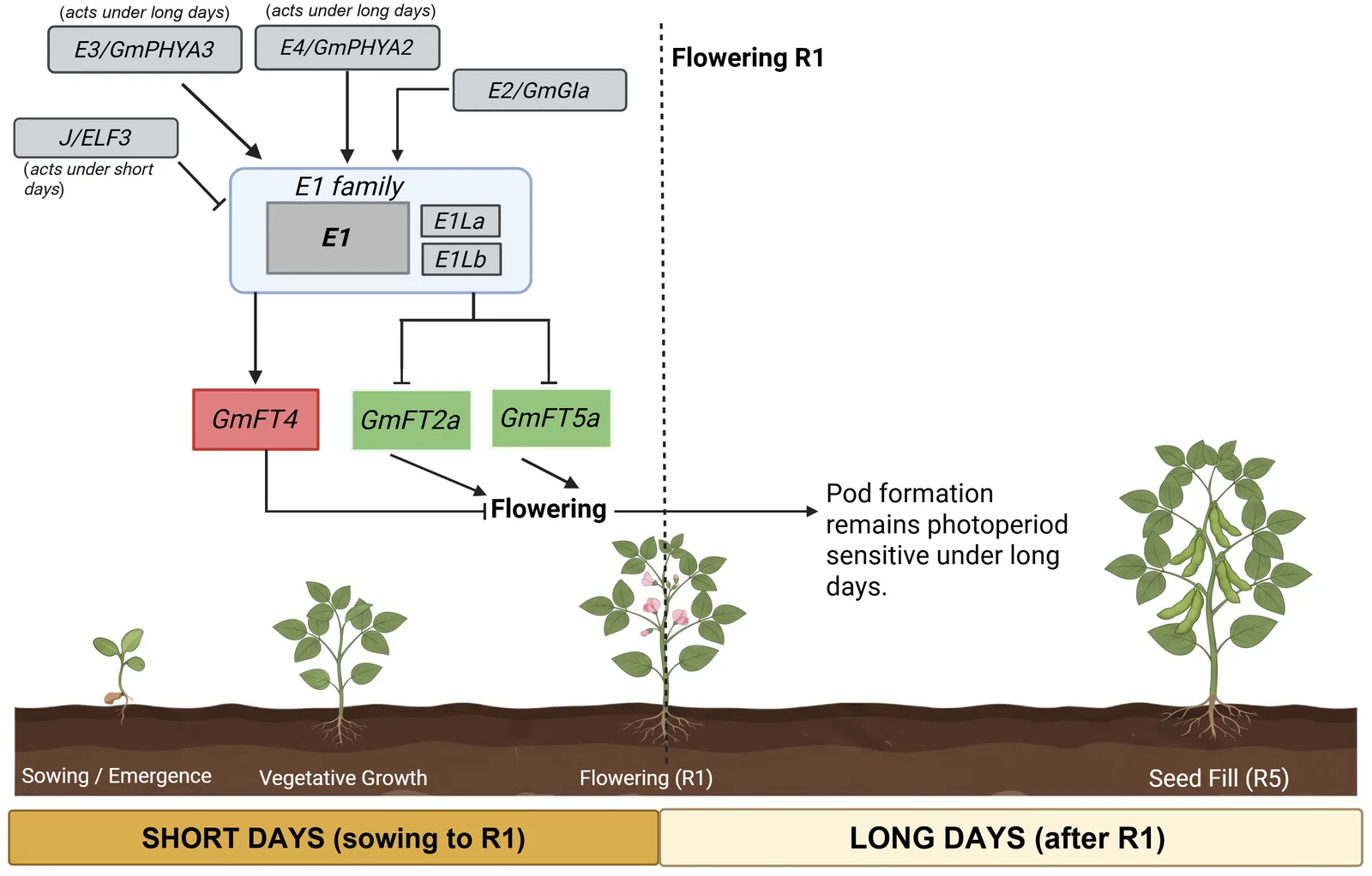
Photoperiod control of flowering in soybean. The network shows regulatory connections whose activity depends on photoperiod; the bands below the developmental timeline mark the photoperiod applied before and after R1 in stage-dependent protocols. Under short days, *E1* is suppressed, and the florigens *GmFT2a* and *GmFT5a* promote flowering; under long days, *E1* is induced and represses them, while activating the repressor *GmFT4*. *E3* and *E4* encode phytochrome A photoreceptors, so their input to the *E1* family depends on photoperiod and light quality; *E2* also acts upstream of the *E1* family (Jia et al., 2025), and *J/ELF3* represses *E1* under short days. Photoperiod sensitivity persists after flowering, with pod formation delayed under long days.

Temperature effects cannot be considered independently of photoperiod. In 91 cultivars (MG 000–VIII) grown under different photoperiod and temperature conditions, flowering time was mainly controlled by additive-by-environment and epistasis-by-environment interactions (Mao et al., 2017). Soybean shows contrasting flowering responses to elevated temperature under short days, flowering earlier as temperature rises from 25 to 30°C but later at 35°C (Tang et al., 2023). The acceleration at 30°C occurred in all cultivars tested, but the delay at 35°C depended on genotype and was almost absent in the two early-maturing cultivars (MG 0 and I). The *phyA–E1* pathway mediates the delay at 35°C, whereas the acceleration at 30°C involves up-regulation of *GmFT2a* and *GmFT5a* and is independent of *E1* (Tang et al., 2023). In a scan for quantitative trait locus (QTL)-by-environment interactions, a maturity QTL with *GmAPETALA1d* as the candidate gene showed reversed allelic effects in one of three years, possibly owing to differences in precipitation or temperature (Zhu et al., 2023).

The photoperiod responses described above define the stage-dependent photoperiod window, the interval during which an extended photoperiod can be applied without suppressing floral induction. Long days delay flowering through *E1*-mediated repression of *GmFT2a* and *GmFT5a*, whereas in one study a 22-h photoperiod applied from R1 accelerated seed maturity (Aciksoz et al., 2025). The window, therefore, opens at R1, and its boundaries are genotype dependent. Before R1, the photoperiod must remain below the critical photoperiod, which declines from 16.4 h in MG 0000 to 13.4–13.7 h in MG V–VIII and to 12 h or less in MG IX–X (Yang et al., 2019). A 12-h day, commonly used as the short-day treatment, therefore does not delay flowering in MG 0000–VIII but may do so in MG IX–X. Allelic state at the maturity loci determines both the strength and timing of the response. Plants carrying the loss-of-function alleles described above flower earlier under long days. A single switch point cannot, therefore, be assumed across maturity groups.

This genotype dependence has three implications for rapid generation advancement. First, because allelic variation at the *E1–E4* loci accounts for much of the variation in flowering time (Miladinović et al., 2018), genetically diverse materials may require different photoperiod conditions to achieve rapid cycling. Second, photoperiod sensitivity extends beyond flowering, and long days can delay pod set through the same *E1*-family and Evening Complex genes that control flowering (Sun et al., 2024). Third, because the same loci also contribute to adaptation across latitudes, accelerated cycles could shift allele frequencies at the loci that determine where a line is adapted.

## 3 Rapid generation advancement strategies in soybean

### 3.1 Controlled-environment speed breeding

Speed breeding reduces generation time by manipulating growth conditions. It integrates well with MAS and high-throughput phenotyping (HTP) for multi-trait selection (Wanga et al., 2021). In soybean, the key target is flowering time, governed by photoperiod and temperature: short days and the higher temperatures of summer sowing additively accelerated the transition from emergence to first flowering (Mao et al., 2017). Greenhouse and growth-chamber protocols regulate photoperiod, light quality, and intensity (Ghosh et al., 2018). Such systems allow up to five generations per year in soybean, compared with one or two in the field or greenhouse (Nagatoshi and Fujita, 2019; Jähne et al., 2020). Most soybean protocols use a short photoperiod while optimizing light quality and intensity. Of the maturity loci, *E1* is the most sensitive to the red-to-far-red ratio, *E4* intermediate and *E3* the least (Cober et al., 1996).

LED-based protocols achieve similar generation rates under different lighting regimes. A 10-h photoperiod under a blue-enriched, far-red-deprived light spectrum induced flowering at approximately 23 days and maturity within 77 days (Jähne et al., 2020). Under a 9-h photoperiod, 28 varieties were compared across two light sources, three light conditions and two planting densities. LED light at a photosynthetic photon flux density (PPFD) of 506 μmol m^-^² s^-^¹ at 30 cm, with 5 × 5 cm spacing, 25 ± 2°C and 50 ± 10% humidity, was optimal: a generation averaged 73 days, allowing five generations per year (Lee et al., 2023).

A stage-specific photoperiod has given the highest reported number of generations per year. Under cool-white (6000 K) and red (660 nm) LEDs at 550 μmol m^-^² s^-^¹ at canopy level, plants were grown under a 12-h photoperiod until flowering initiation (R1, ~24 days after sowing), after which the photoperiod was extended to 22 h. This increased the germination of green seed harvested 58 days after sowing from 44% under a continued 12-h photoperiod to 92%. Combined with a dormancy-breaking treatment of the green seed, the protocol gave a 63-day cycle, close to six generations per year, although the cycle was compared with field and standard greenhouse or growth-chamber cycles, and no cycle length was given for the 12-h treatment (Aciksoz et al., 2025).

Light quality also affects cycle length. Under an 8-h light/16-h dark cycle, red–white LEDs at 513 μmol m^-^² s^-^¹ shortened flowering to 29–33 days and maturity to 66–72 days, reducing generation time by 24–31 days relative to approximately 91–103 days in the field. Blue LEDs at 231 μmol m^-^² s^-^¹ gave 33–38 days to flowering and 90–95 days to maturity (Masangano et al., 2025). However, the spectral comparison is confounded, because the blue treatment was run at less than half the intensity and in a different chamber.

Temperature and CO_2_ availability interact with light to influence plant growth and development. Controlled-environment protocols combine temperature and photoperiod regimes to promote rapid growth and earlier flowering (Wanga et al., 2021). In eight cultivars grown in pots in open-top chambers, elevated CO_2_ increased seed yield by 40% and shoot biomass by 46% on average, though the yield response differed among cultivars (Li et al., 2017). These responses may not represent those of field-grown soybean. Under fluorescent lamps at 220 μmol m^-^² s^-^¹, a 14-h light (30°C)/10-h dark (25°C) cycle and CO_2_ above 400 ppm reduced generation time in the Japanese soybean cultivar Enrei from 102–132 days in the field to 70 days (Nagatoshi and Fujita, 2019). At the same intensity and CO_2_ level, a 10-h light (30°C)/14-h dark (25°C) cycle reduced the time to flower initiation in Williams 82 from 40–60 days to 28.0 days. CO_2_ supplementation promoted growth and yield without reducing days to flowering, and increased healthy flower number from 10.9 to 32.0 over the first five days of flowering, improving crossing efficiency (Nagatoshi and Fujita, 2019). Flowering responses to elevated CO_2_ vary with growth conditions and genotype (Springer and Ward, 2007).

Not every rapid protocol holds plants under short days before flowering, and the clearest exception is consistent with a genotype-dependent photoperiod window. In the CO_2_-supplemented protocol above, Enrei, a weakly photosensitive cultivar, was grown under the longer photoperiod, and the authors considered that the photoperiod needs to be adapted for each cultivar (Nagatoshi and Fujita, 2019). A low-cost chamber protocol for the long-duration cultivar DS9712 applied a 16-h light/8-h dark photoperiod from emergence to V4. Chamber-grown plants completed flowering in 31 days against 46 days in the control, but pod formation took longer, days to maturity did not differ (125.7 against 125.3 days), and seed yield per plant was lower (4.2 against 6.9 g) (Rajendran et al., 2025). Earlier flowering therefore did not shorten the generation, and it may also reflect the effect of temperature, because the control grew at an average of 37.5/27°C in July against about 30/20°C in the chamber, and high temperature can delay soybean flowering (Tang et al., 2023). As DS9712 is described only as a photoperiod-sensitive, long-duration cultivar, without its maturity group or E-locus alleles, the result cannot be related to its critical photoperiod.

Plant density can be increased under speed breeding to advance large single-seed-descent populations, although the appropriate sowing density must be determined for each setup and end use (Ghosh et al., 2018). Rapid generation advancement therefore depends on optimizing photoperiod, light quality and intensity, temperature, CO_2_ and planting density together, while accounting for genotype-specific flowering responses. The principal environmental factors and breeding outcomes of controlled-environment soybean speed breeding are summarized in **Figure 2**.

**Figure 2:**
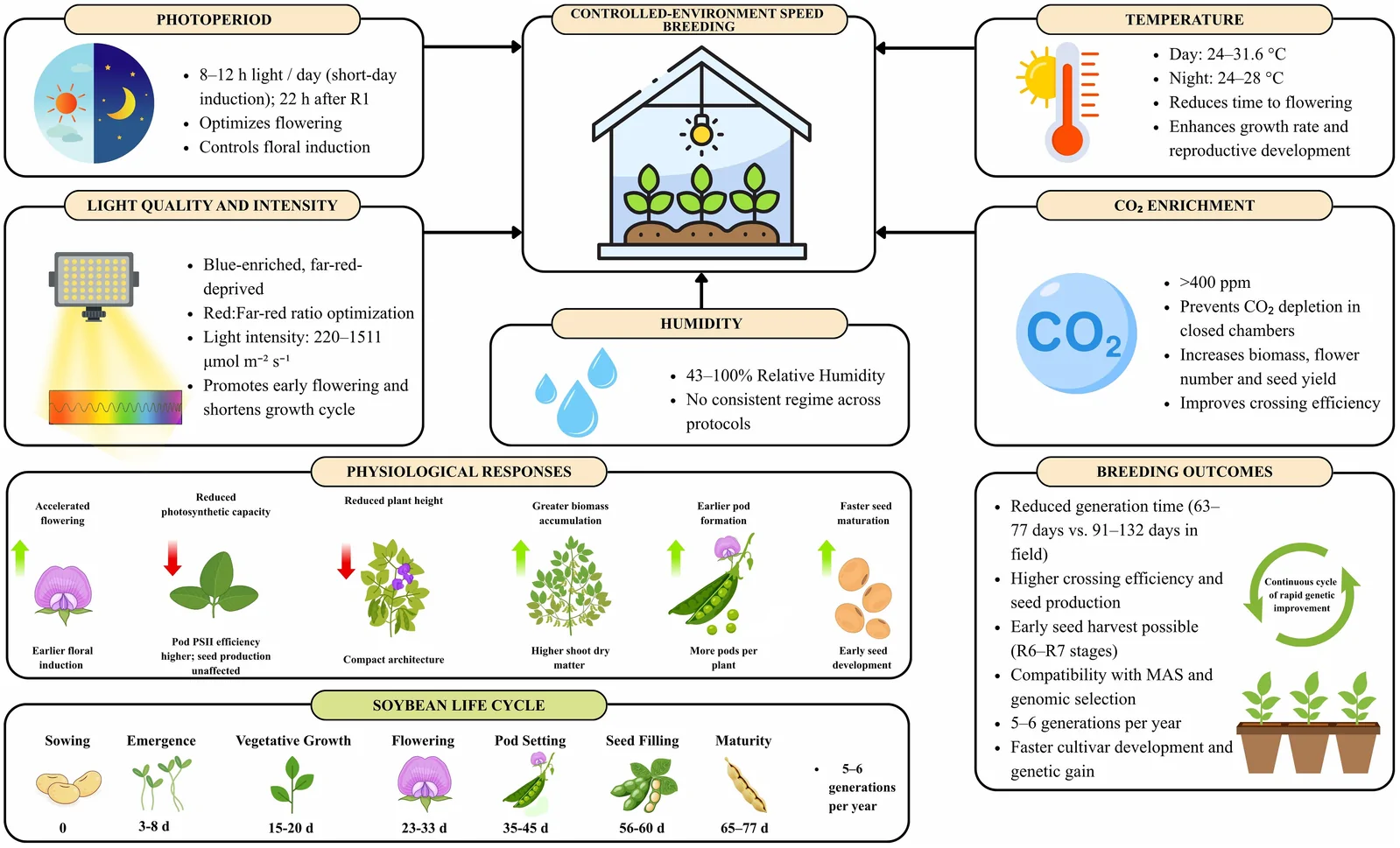
Environmental control in soybean speed breeding. Photoperiod, light quality and intensity, temperature, CO_2_ and humidity are manipulated together. Parameter ranges are compiled from published soybean protocols, and life-cycle timings are a synthesis across them.

### 3.2 Complementary generation-advancement approaches

Several complementary strategies can shorten breeding cycles or increase generations per year in soybean. Single-seed descent integrates readily with speed breeding, advancing segregating populations toward homozygosity by retaining one seed per plant per generation (Wanga et al., 2021). In rice, seed-based sampling improves MAS efficiency in such programs while reducing sampling costs (Arbelaez et al., 2019). Harvesting before full maturity shortens the cycle by reducing pod maturation and seed drying time. When immature embryos were cultured, plantlets were obtained from 73% of those that germinated, and cycle length was reduced from 130–140 to 65–70 days (Roumet and Morin, 1997). Early harvest can be combined with controlled-environment protocols. Pods harvested at R6 and oven-dried for five days at 37°C and 10–20% relative humidity gave 83.33–100% germination (Masangano et al., 2025).

Where controlled infrastructure is unavailable, off-site and off-season nurseries exploit favorable natural conditions. Off-site summer nurseries produced two generations of Northeastern Spring and Yellow–Huai–Hai cultivars in Beijing and Hainan, respectively, compared with one at the origin. Fresh seeding saved 7–10 days and supported at least four generations annually, while *E1–E4* haplotypes were associated with regional adaptation (Fang et al., 2021). Doubled haploid technology achieves full homozygosity in two generations, compared with six to eight generations required by self-pollination. However, its use in soybean is constrained by low induction rates and hybridization barriers, with progress dependent on genome editing and tissue culture (Liu et al., 2025). These approaches share with controlled-environment speed breeding the objective of faster generation turnover and homozygosity. They can be combined with MAS, and their effectiveness depends on genotype- and environment-specific responses. The principal soybean generation-advancement strategies and reported cycle lengths are compared in **Table 1**.

**Table 1:** Comparison of principal soybean generation-advancement strategies and reported cycle lengths.

Reference	Strategy	Photoperiod and light (PPFD, μmol m^-^² s^-^¹)	Temperature (day/night), RH, CO_2_	Genotypes (MG)	Cycle (d)	Generations per year
(Aciksoz et al., 2025)	Long photoperiod after floral induction	12 h to R1 (~24 DAS), then 22 h; cool-white and red LEDs, 550	24/24°C; RH 80–90%; no CO_2_ supplementation	Not reported	63	6 (calculated)
(Jähne et al., 2020)	Short photoperiod, blue-enriched spectrum	10 h; blue-enriched, far-red-deprived LEDs, ~500	28/28°C; RH 80–100%	8 genotypes (MG 000–V)	77	5 (calculated)
(Lee et al., 2023)	Indoor LED protocol	9 h; LEDs, 506 at 30 cm	25 ± 2°C; RH 50 ± 10%	28 varieties (early to late maturing)	73	5 (calculated)
(Masangano et al., 2025)	Red–white vs blue LEDs	8 h; red–white LEDs 513, blue LEDs 231	31.6/24.5°C; RH 43/57%	4 accessions, each of a different MG (not specified)	66–72	Not reported
(Harrison et al., 2021)	Red–blue vs full-spectrum LEDs	12 h; red–blue 1018, full-spectrum 1190	29/27°C	2 lines (MG 4.7, 5.6), an F3 population, 16 introductions (MG 00–9)	63–81 (mid and late maturities)	Up to 5 (calculated)
(Karaman et al., 2026)	Constant photoperiods × LED spectra	Constant 10, 14, 18 or 22 h; four LED spectra, 400–600	28 ± 2°C; RH ≥ 50%; CO_2_ 524–587 ppm, not supplemented	2 cultivars (about MG III–IV)	86 (to R8, at 10 h)	Not reported
(Nagatoshi and Fujita, 2019)	CO_2_ supplementation, cultivar-specific photoperiod	14 h (Enrei) or 10 h (Williams 82, BR16); fluorescent lamps, 220	30/25°C; RH 50–80%, not regulated; CO_2_ above 400 ppm	3 cultivars (MGs not stated); full cycle in Enrei only	70	5 (calculated)
(Taku et al., 2024)	Short photoperiod with and without CO_2_ supplementation; harvest at R7 (vegetable soybean)	10 h; fluorescent tubes, 220	30/25°C; RH 70–80%; CO_2_ 550 ppm or ambient	16 genotypes (MG not reported)	81–92 (with CO_2_)	4–4.5 (calculated)
(Gallino et al., 2022)	Converted cold-storage chamber, harvest at late R5	12 h; cool-white fluorescent, 350	24°C	7 F7 lines (MG late IV to late VI)	60	Up to 5 (five successive cycles run)
(Fang et al., 2021)	Off-site nursery with fresh seeding and MAS	Natural daylength; off-site summer nurseries, glasshouse in winter	Ambient; glasshouse in winter	1 cultivar, Heihe27 (early maturing)	~73	5 (achieved)
(Roumet and Morin, 1997)	Immature embryo culture	Greenhouse; natural light supplemented with sodium-vapour lamps (15 h)	15–25°C (greenhouse)	1 cultivar, Kingsoy (MG II)	65–70	Up to 5 (calculated)

DAS, days after sowing; MAS, marker-assisted selection; MG, maturity group; PPFD, photosynthetic photon flux density; RH, relative humidity. Generations per year are marked as calculated when derived from a single cycle length, and as achieved when demonstrated over successive generations.

## 4 Supporting techniques and technologies for RGA

Soybean RGA lies at the interface between agronomy and controlled-environment engineering. Precise environmental control depends on appropriate facilities and equipment. This section covers growth facilities and lighting, climate control, root-zone management, and sensor-based phenotyping. **Figure 3** combines them into one workflow of environmental control, non-destructive imaging, early selection and rapid recycling of immature seed, and highlights the parts that are not yet demonstrated in soybean.

**Figure 3:**
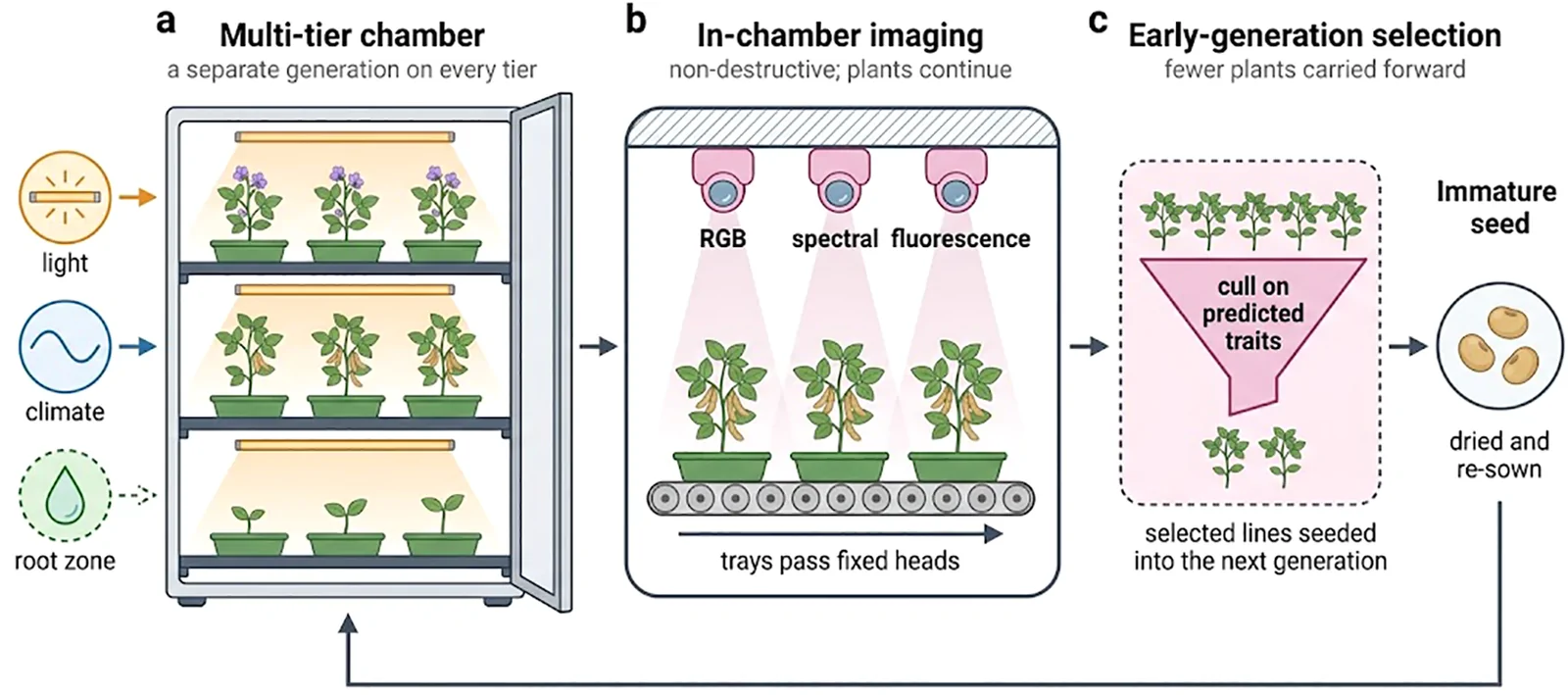
Supporting hardware for RGA in soybean. **(a)** Multi-tier growth chamber integrating control of light, climate, and root-zone conditions. LED lighting provides approximately 500 µmol m^-^² s^-^¹ PPFD, while temperature and CO_2_ are controlled during plant growth. Holding a different generation on each tier requires each tier to be lit independently and shielded from the light of the others, because, in stage-dependent protocols, plants before and after R1 are held under different photoperiods (Section 2). The dashed root-zone connection marks hydroponic control as a proposed component of soybean RGA. **(b)** In-chamber non-destructive imaging using RGB, spectral and chlorophyll-fluorescence sensors to assess plant architecture, pigment-related traits and photosystem II efficiency. **(c)** Conceptual early-generation selection based on predicted traits, allowing fewer plants to be advanced. The dashed outline indicates that sensor-based selection has not yet been demonstrated within a soybean RGA chamber. An immature seed can then be dried and re-sown to shorten generation turnover. Solid elements represent components demonstrated in soybean, whereas dashed elements indicate proposed or not yet demonstrated applications. Abbreviations: CO_2_, carbon dioxide; PPFD, photosynthetic photon flux density; RGA, rapid generation advancement; RGB, red–green–blue.

A 0.225 m² benchtop cabinet holds eight 1 L pots and consumes 6.24 kWh per day, making it suitable for small-scale pilot trials before scale-up (Ghosh et al., 2018). Chambers of ~0.4 m³ could hold at least 30 plants in 0.4 L pots (Nagatoshi and Fujita, 2019), and 12 speed-breeding boxes fit within one climate chamber, enabling parallel testing (Jähne et al., 2020). Larger facilities include LED-supplemented glasshouses of 30 and 66.4 m² (Ghosh et al., 2018) and a low-cost LED growth room used for wheat, barley, oat and triticale (Watson et al., 2018). No single facility architecture is standard for soybean RGA; systems are adapted to experimental scale and breeding objective. Planting density also matters, as 96-cell plates of 75 cm³ allow ~750 plants per cubic meter at a seed-to-seed turnover of ~75 days (Jähne et al., 2020).

Growth chambers start at tens of thousands of dollars (Ghosh et al., 2018). In a vertical farming system, soybean was estimated to yield about eight times more protein per m² per year than in the open field. Meeting one adult’s annual protein requirement (21 kg) would take 20 m² of crop area and 2.4 m³ of water but 16 MWh of electricity, compared with 164 m², 111 m³, and 0.009 MWh in the field (Righini et al., 2024). A minimum indoor production cost of ≈$10 per kg of dry matter was estimated from energy use in vertical farming (Lovat et al., 2025). Both estimates refer to food production, whereas in breeding the objective is generation advancement. Programming the dark period to coincide with peak tariff periods can provide substantial savings (Ghosh et al., 2018).

Because soybean is a facultative short-day species, light imposes greater constraints on RGA than in long-day cereals. Several LED-based soybean protocols use about 500–550 μmol m^-^² s^-^¹ (Jähne et al., 2020; Aciksoz et al., 2025), whereas fluorescent-lit chambers have used around 220 μmol m^-^² s^-^¹ at the canopy level (Nagatoshi and Fujita, 2019). A protocol for long-day and day-neutral crops recommended ~450–500 μmol m^-^² s^-^¹, but soybean was not among the species tested (Ghosh et al., 2018). Increasing intensity above 1000 μmol m^-^² s^-^¹ advanced flowering by only two days compared with 500–900 μmol m^-^² s^-^¹ (21.8 ± 1.1 versus 23.9 ± 1.1 days), a modest gain for the added energy cost (Jähne et al., 2020). Light quality also affects plant architecture. A lower red-to-blue ratio gave shorter, sturdier plants without affecting flowering time, while blue light alone gave no further height reduction (Jähne et al., 2020). Light-quality responses are species- and system-dependent. Reducing the red-to-far-red ratio from 5.6 to 3.1 or below accelerated lentil flowering by 10–11 days *in vitro* (Mobini et al., 2016), whereas in soybean, far-red light did not accelerate flowering and induced petiole elongation and lodging (Jähne et al., 2020). The difference in cultivation systems limits direct comparison.

Day temperatures across the controlled-environment protocols range from 24 to 31.6°C (**Table 1**). Relative humidity varies more widely, from 43 to 100%, and is not always regulated (**Table 1**). CO_2_ management matters in enclosed chambers, where photosynthetic demand depletes CO_2_ during the light period. At 25 days after flowering, the unsupplemented chamber held ~200 ppm CO_2_ under light and ~750 ppm in darkness, compared with 400–600 ppm and ~1,200 ppm with supplementation (Nagatoshi and Fujita, 2019). Supplementation increased seed number per plant from 26.6 ± 2.5 to 53.4 ± 9.1, and average seed weight was also significantly higher, with no significant difference in seeds per pod (Nagatoshi and Fujita, 2019).

Soybean protocols rely predominantly on soil- or substrate-based systems. These include a turf–wood-fiber–clay substrate mixed with soil and inoculated with rhizobia (Jähne et al., 2020), commercial potting soil (Aciksoz et al., 2025), germination on vermiculite followed by a soil mixture (Nagatoshi and Fujita, 2019), and a sand–cocopeat–vermiculite mixture (Taku et al., 2024). Rapid vegetative growth also increases the risk of nutritional disorders. In the protocol for long-day and day-neutral crops, tip-burn necrosis from calcium deficiency is managed with a 1% (w/v) calcium nitrate foliar spray two to three times per week (Ghosh et al., 2018). Root-zone conditions have received far less attention than above-ground variables; hydroponics could provide independent control of root-zone temperature, electrical conductivity, pH, and oxygen. In a hydroponic system, root-zone temperature control increased baby-leaf vegetable yield under extreme weather, but not under mild conditions (Karnoutsos et al., 2021).

To our knowledge, no study has directly compared hydroponic and soil-based systems for generation time in soybean. In pea, an *in vivo* protocol achieved 5.3 generations per year using vermiculite hydroponics with scheduled fertilization, the plant growth regulator flurprimidol (0.6 μM), a 20-h photoperiod, 21/16°C, 500 μmol m^-^² s^-^¹, and 266 plants m^-^² (Mobini and Warkentin, 2016). The root-zone contribution cannot be isolated from the co-applied treatments, so hydroponics is a plausible option for soybean RGA but has not been demonstrated.

Early harvesting and germination of immature seed further shorten the seed-to-seed cycle. Pods harvested 37 days after flowering and air-dried for eight days gave 100% germination within seven days, contributing to a 70-day cycle that also depended on CO_2_ supplementation and optimized light and temperature (Nagatoshi and Fujita, 2019). In pigeonpea, 35-day-old seeds gave 100% germination, and germinating immature seeds allowed three to four generations per year (Saxena et al., 2017). Embryo culture achieves comparable cycle lengths, so direct germination is preferred for its simplicity. Embryo rescue requires dissection, culture medium, and sterile conditions (Roumet and Morin, 1997), whereas the protocol of Jähne et al. (2020) achieved a cycle of 77 days without *in vitro* culture. Embryo rescue also serves a distinct objective. In wide crosses with wild *Glycine* relatives, it is used to recover viable hybrid progeny. From 90 rescued embryos, about 37% developed into plantlets *in vitro* and 8% into mature plants, yielding seven confirmed hybrids (Bodanese-Zanettini et al., 1996).

Speed breeding accommodates only a limited number of crosses and segregating populations (Kayess et al., 2025). HTP could alleviate part of this constraint without increasing chamber capacity. **Figure 3b** shows an imaging station, with trays moving through fixed RGB (red, green, blue), spectral, and chlorophyll-fluorescence sensors. Chlorophyll-fluorescence imaging has been applied to intact soybean plants, but required 1 h of dark adaptation, a fixed 9 a.m.–3 p.m. window, and transfer of the plants to an imager at room temperature (Aciksoz et al., 2025). A platform integrating hyperspectral reflectance, RGB imaging and nighttime chlorophyll fluorescence has been evaluated in soybean (Hassan and Chang, 2025), though its fluorescence measurements are restricted to the dark period.

Sensor-derived indices can be sensitive to the growth environment. Leaf F_v_/F_m_ decreased significantly from 0.704 under a 10-h photoperiod to 0.400 under a post-flowering 22-h photoperiod, whereas pod F_v_/F_m_ was unaffected at 0.781 and 0.780 (Aciksoz et al., 2025). To our knowledge, sensor-based early-generation selection has not yet been implemented within a soybean RGA system; the closest work imaged soybean under controlled conditions without using the resulting traits as selection criteria (Hassan and Chang, 2025). Early culling based on predicted traits, which would increase throughput within a fixed growth area, therefore remains a projected benefit of HTP (**Figure 3c**).

Chambers impose a defined selection environment, and whether plants selected under ~500 μmol m^-^² s^-^¹ and an abbreviated reproductive phase perform comparably in the field remains uncertain (Section 5). Genotyping can complement selection in the chamber, and selection for early-maturity alleles at soybean E loci is already used in field-based breeding programs (Yerzhebayeva et al., 2023).

## 5 Physiological and developmental limitations of accelerated generation advancement

Acceleration is not physiologically neutral, because each manipulation that shortens generation time also alters the plant it produces. Breeders need to identify the changes relevant to subsequent selection. A treatment may injure plants sufficiently to compromise seed supply, influence which genotypes are advanced to the next cycle, or change the ranking of genotypes for a given trait. The first effect is comparatively well characterized. The second applies to every application, including chambers used purely for multiplication. The third applies only where phenotypes are scored under accelerated conditions. In soybean, the last two have received little study (Section 5.4).

### 5.1 Physiological costs and seed production constraints

Extending the photoperiod imposes a measurable physiological cost on soybean. In plants grown under a 10-h photoperiod until R1, extending the photoperiod to 22 h reduced leaf Amax, Vcmax, and Jmax by 37%, 55% and 40%, respectively, relative to plants kept at 10 h (Aciksoz et al., 2025). Pod photosystem II operating efficiency was higher under 22 h, and seed dry biomass was not reduced. The authors proposed that the increased pod photosynthetic activity may contribute to the higher germination of immature seed (Aciksoz et al., 2025). Continuous-light injury is documented in tomato, eggplant, potato, and other species (Velez-Ramirez et al., 2011), with tolerance in tomato mapping to a single locus (Velez-Ramirez et al., 2014). In *Arabidopsis*, abrupt photoperiod extension also triggers an oxidative burst-like response, with increased apoplastic peroxidase and decreased catalase activity (Abuelsoud et al., 2020). Prolongation of the light period is recognized as a photoperiod stress syndrome (Roeber et al., 2021, Roeber et al., 2022).

The magnitude of injury depends on light intensity (Velez-Ramirez et al., 2011), so light exposure can be adjusted. In lentil under an 18-h photoperiod, 148–167 μmol m^-^² s^-^¹ caused high stress, and one of two varieties died before flowering, whereas lower intensities (74–129 μmol m^-^² s^-^¹) gave flowering at 26–31 days (Mitache et al., 2024). Spectrum is another factor, as red–blue light reduced plant height and shortened the period from planting to maturity by 1.5 d compared with full-spectrum light, without affecting vegetative biomass, pods, or seeds per plant (Harrison et al., 2021).

Besides delaying pod formation, long days after flowering caused abscission of the first wave of flowers (Sun et al., 2024). In the same study, pollen germination and pollen-tube length did not differ significantly between photoperiods, but long days (16 h) altered pistil development. Under short days (12 h), the stigma formed a curved, hook-like apex and reached the height of the anthers when pollen was shed, whereas under long days the hook was less pronounced and the stigma stood above the anthers, an arrangement the authors considered less conducive to rapid pollination (Sun et al., 2024).

Seed supply is a main operational constraint and is well characterized. In 96-cell trays, seed number per plant was reduced, although plants still produced on average more than five seeds, enough for single-seed descent (Jähne et al., 2020). Germination of immature soybean seed increased with harvest date, from ~50% at 56 days to ~90% at 77 days after sowing, and with light intensity, from ~60% at 480 to ~80% at 1511 μmol m^-^² s^-^¹ at 63 days (Jähne et al., 2020). More importantly, seeds harvested early showed substantial genotypic variation in germination, whereas germination became more consistent with delayed harvest (Jähne et al., 2020). Applying the same early harvest date to all plants may therefore gradually change the genetic composition of the population.

### 5.2 Controlled environments for generation advancement and seed multiplication

Chambers used only for multiplication should be considered separately. When accelerated conditions serve only to advance generations and selection takes place later in the field, chamber phenotypes are not used for selection. Their correlation with field performance is therefore not relevant. However, this applies only if generation advancement does not depend on genotype, which cannot be assumed. In chickpea, generation cycles spanned 43–60 days in early-maturing accessions, 44–64 days in medium-maturing accessions, and 52–79 days in late-maturing accessions (Samineni et al., 2020). One late-maturing accession could not be advanced beyond three cycles because of its response to photoperiod and temperature (Samineni et al., 2020). In soybean, generation advancement may also depend on genotype because germination of early-harvested seed varies among genotypes.

A further risk in soybean is that photoperiod responses are controlled by the same regulatory network involved in environmental adaptation. These responses therefore depend on genetic background, even after flowering (Section 2). During repeated accelerated cycles, a segregating population is exposed to conditions that may affect reproductive development, fertility, and seed maturation differently among *E1–E4* haplotypes. Differences in survival and fertility, combined with fixed harvest dates, could shift allele frequencies. As a result, the F6 population entering the field could differ systematically from the F2 population entering the chamber, especially at loci involved in latitudinal adaptation. To our knowledge, no soybean study has measured these shifts under current controlled-environment RGA protocols. However, Martin et al. (1978) found that single-seed descent in the greenhouse at high plant density shifted allele frequencies for flower and pubescence colour. The resulting progenies also matured 0.5–3.6 days later than those advanced at low density.

### 5.3 Controlled environments for phenotypic selection

When selection is performed on chamber-grown plants, selection efficiency also depends on the genetic correlation between traits measured in the chamber and in the field. Although trait means often differ between these environments, the important question is whether the ranking of genotypes is similar in both. An analysis of 17 experiments found a modest median r² of 0.26 between laboratory and field phenotypic data (Poorter et al., 2016). However, the median r² for yield of the same genotypes grown at the same field site in two consecutive years was significantly lower, at 0.08 (Poorter et al., 2016). Several differences between controlled and field conditions may influence plant performance. The photothermal ratio was consistently lower under controlled conditions, which may affect the source–sink ratio (Poorter et al., 2016). Plant density also differs, and a meta-analysis found that doubling density reduced total vegetative mass per plant by a median of 33%, with strong negative effects on side-shoot formation but little effect on plant height (Poorter et al., 2016). Pots provide a much smaller rooting volume than plants typically have in the field (Poorter et al., 2016). In soybean plants grown individually in pots in a chamber, root restriction likely contributed to lower vegetative growth and reproductive output than in field-grown plants (Karaman et al., 2026). Genotype × environment interactions remain a challenge, as traits selected under controlled speed-breeding conditions often fail to translate consistently across field environments (Kayess et al., 2025).

In wheat, correlations between traits measured under speed-breeding and field conditions vary by trait. In a panel of 21 spring wheat genotypes, adult-plant responses to leaf rust under speed-breeding conditions correlated with field measurements, with R² values ranging from 0.43 to 0.77 depending on the leaf assessed and the field assessment date (Riaz et al., 2016). Across 609 genetic resources and 184 recombinant inbred lines, days to heading (r = 0.691) and culm length (r = 0.854) were correlated between a modified speed-breeding system and the field (Cha et al., 2023). Within the recombinant inbred lines alone, these correlations were weaker, at r = 0.248 and 0.425, respectively (Cha et al., 2023). Neither awn length nor spikelet number per spike showed a significant correlation between the two conditions. All lines selected for early heading and short culm length under speed breeding headed earlier in the field than the late-heading parent (Cha et al., 2023). Plant height in spring wheat showed consistently strong genetic correlations between speed-breeding and field conditions (Watson et al., 2019). The predicted efficiency of indirect selection for height under speed breeding was 66–100% of that of direct selection in the field, whereas the expected response to indirect selection for grain yield was low (Watson et al., 2019). Selection efficiency also varies among field environments. In winter wheat grown under different nitrogen and fungicide treatments, the efficiency of indirect selection for grain yield relative to direct selection ranged from 0.15 to 0.99 (Brancourt-Hulmel et al., 2005). Indirect selection was never more efficient than direct selection (Brancourt-Hulmel et al., 2005).

### 5.4 Knowledge gaps in the physiological and developmental responses of soybean

The soybean protocols in **Table 1** report cycle lengths, but none reports genetic correlations between chamber and field traits, realized genetic gain in the field following chamber-based selection, or changes in allele frequency over successive cycles. To our knowledge, no other soybean study using current controlled-environment RGA protocols has reported them either. In cereals, selection under speed-breeding conditions has been evaluated in the next generation and in the field. In durum wheat, a weighted selection index applied under speed breeding shifted the population mean for four of five traits, as shown by comparing selected and unselected F3 families grown under the same conditions (Alahmad et al., 2018). In barley, screening for multiple traits under speed breeding produced introgression lines whose disease resistance was subsequently confirmed in field screening nurseries in Australia and Uruguay (Hickey et al., 2017).

Because advancement may depend on genotype even when the chamber is used only for multiplication, soybean populations should be genotyped before and after accelerated advancement, at least at the major maturity loci. This would show whether allele frequencies change. Phenotyping the same population under accelerated and field conditions would give the genetic correlations between chamber and field traits. Seed composition should not be selected on chamber-grown plants without this validation, because environmental conditions during seed development affect the accumulation of protein and oil in the seed (Rotundo and Westgate, 2009). Until these measurements are made, the evidence supports soybean RGA as a method for accelerating generation turnover, and phenotypic selection in the chamber needs to be validated in the field.

## 6 Integration of RGA with selection and genomic tools

RGA shortens the breeding cycle, but it still requires a method of selection. Traits measured in the chamber may not predict performance in the field, and for soybean the correlation between the two has not been reported (Sections 5.3 and 5.4). Genotypic selection depends less on how traits are expressed in the chamber. This section covers the integration of RGA with MAS for loci with known effects, genomic selection for quantitative traits, and genome editing for targeted modifications.

MAS can easily be incorporated in a controlled-environment speed-breeding system (Jähne et al., 2020). Where a trait is controlled by one or a few loci of large effect, plants can be genotyped at the seedling stage, and only carriers of the required alleles are advanced, without waiting for the phenotype. In the off-site nursery system described in Section 3.2, DNA markers for the maturity genes *E1–E4* were used to predict the maturity group and adaptation region of advanced lines, which could save the time needed to phenotype them in the target region (Fang et al., 2021). The same approach could be applied to other loci of large effect, such as major disease-resistance and quality loci, provided that marker–trait associations are consistent across the breeding material. MAS is less effective for yield and other quantitative traits controlled by many loci of small effect (Stewart-Brown et al., 2019). Genomic selection instead uses genome-wide marker information to predict genetic merit of an individual. A training set that is both genotyped and phenotyped is used to estimate marker effects, which then predict genomic estimated breeding values for individuals genotyped but not phenotyped (Stewart-Brown et al., 2019). For RGA, this means that the training phenotypes can come from field trials, while selection among plants in the accelerated generations relies on genomic predictions, without phenotypes from the chamber.

Genomic prediction has been studied in soybean breeding lines. Genotyping by sequencing of 301 elite lines gave a prediction accuracy of 0.64 for grain yield (Jarquín et al., 2014). In 483 elite lines, predictive ability was 0.81 for protein and 0.71 for oil, but 0.26 for yield (Stewart-Brown et al., 2019). Training population size was the factor most relevant to improvement, with the greatest gains in training sets of up to 2,000 individuals, whereas alternative prediction models had minor impacts on accuracy (Xavier et al., 2016). Across maize, soybean, and rice, accuracy improved significantly when only markers significant at *p* ≤ 0.05 or *p* ≤ 0.10 were used, whereas subsets chosen by linkage disequilibrium gave accuracy similar to the full marker set (Kaler et al., 2022). The value of genomic selection in an accelerated pipeline therefore depends on the size of the training population, its relatedness to the selection candidates, and the quality of its phenotypes.

Genomic selection can be effective for quantitative traits such as yield by reducing the duration of the selection cycle, and genomic selection and speed breeding together can reduce the breeding cycle (Watson et al., 2019). For grain yield in a soybean breeding program, genomic selection is expected to give more genetic gain per unit time than phenotypic selection if its cycle length is less than 77% of the cycle length of phenotypic selection (Jarquín et al., 2014). In spring wheat, four traits measured under speed breeding on a 193-line training population and 350 selection candidates were added to multivariate genomic selection models, and the predictive ability for grain yield in the field was significantly increased, in some cases to twice that of univariate models (Watson et al., 2019).

Genome editing makes targeted modifications, but edited material still has to be advanced through several generations before it is useful for breeding. Current genome editing strategies in soybean begin with stable transformation, which delivers a transgene encoding Cas9 and a guide RNA targeting a specific sequence (Michno et al., 2020). Soybean is still recalcitrant to transformation, with low efficiency and genotype specificity, and high-efficiency *Agrobacterium*-mediated transformation is achieved in only a limited number of elite lines (Xu et al., 2022). The transgene in the T0 plant is generally expected to be unlinked to the mutation, so the two should assort independently into the T1 generation, and plants that carry the mutation and no longer carry the transgene can then be selected (Michno et al., 2020). Of four T0 soybean plants tracked into their progeny, only one showed the expected transmission and segregation (Michno et al., 2020). Of the other three, one transmitted neither transgene nor mutations, possibly because the plant was chimeric, one carried four unlinked transgene insertions (two at paralogous target sites), and one transmitted mutations without evidence of transgene integration (Michno et al., 2020). Genotyping in each generation is therefore needed to confirm segregation of the transgene and homozygosity of the edit. RGA can shorten these generations, and so the time needed to recover transgene-free edited lines. Where an edit is made in a genotype chosen for its amenability to transformation, it then has to be introgressed into an elite background by backcrossing, and each of these generations can be run through an accelerated cycle. Markers that measure recovery of the recurrent parent genome allow backcrossing to stop once a high percentage of that genome is restored (Blinkov et al., 2025).

MAS, genomic selection and genome editing can be combined with RGA in a single pipeline (**Figure 4**). A conventional cross or an edited line enters repeated accelerated generations, with selection checkpoints between cycles. At each checkpoint, markers confirm target alleles and, where relevant, loss of the transgene, and genomic prediction ranks lines for quantitative traits. The lines retained then go to multi-environment field trials, because yield is best evaluated in the field and selection for adaptation to the target environment must still take place there (Ghosh et al., 2018). Such integration of speed breeding with high-throughput genotyping, genome editing and genomic selection was envisaged when the protocols were first published (Watson et al., 2018). With RGA, these selection steps can be applied over several generations in the time conventionally needed for one or two generations.

**Figure 4:**
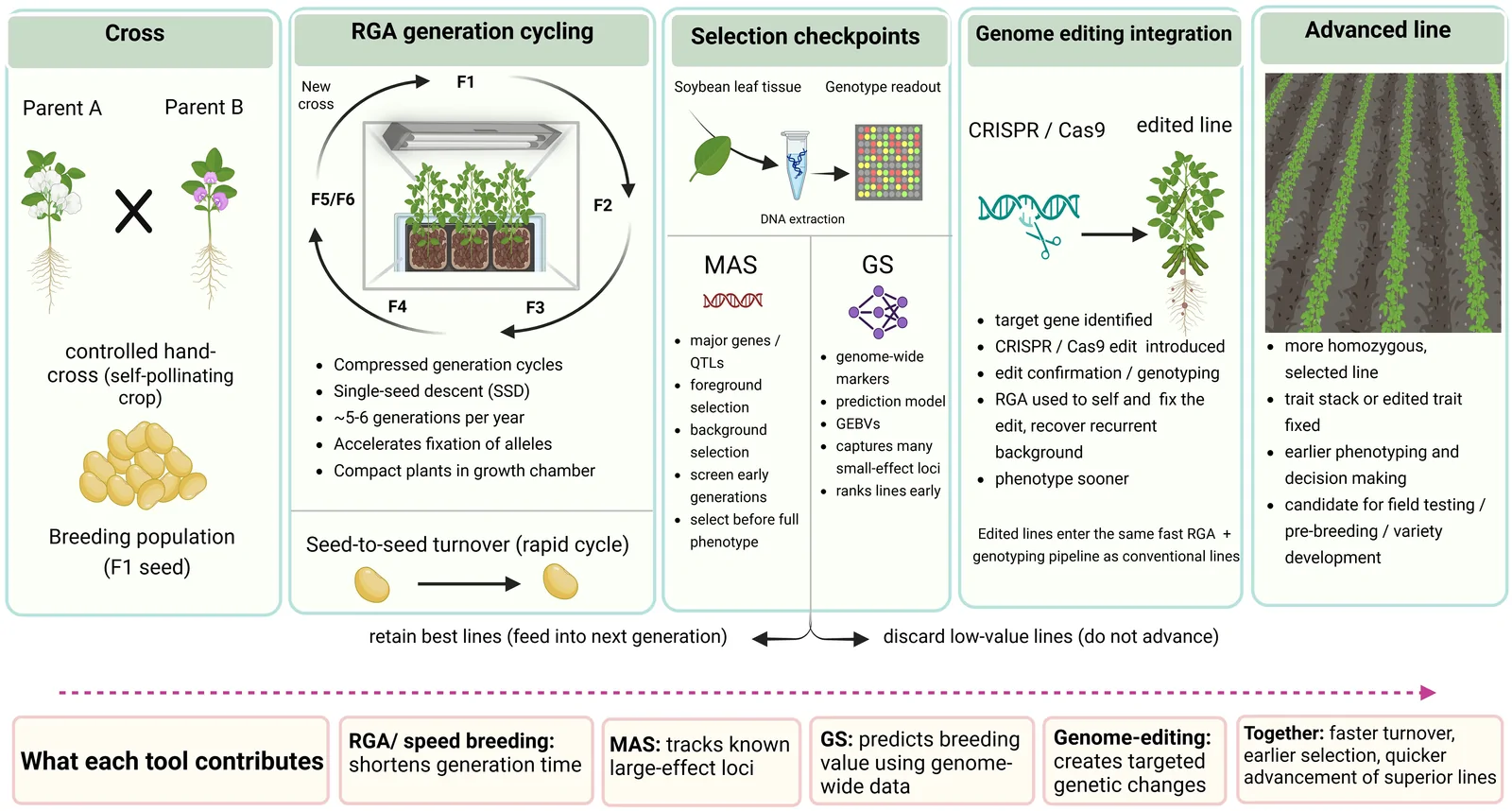
Proposed pipeline integrating RGA with MAS, genomic selection and genome editing. Panels, from left to right, show the cross, generation cycling under RGA, the selection checkpoints at which plants are genotyped for MAS and GS, the entry of edited lines into the same pipeline, and the advanced line that goes to field testing. Arrows below the checkpoints mark the lines retained for the next generation and those discarded. The lower row states what each tool contributes. Abbreviations: GEBVs, genomic estimated breeding values; GS, genomic selection; MAS, marker-assisted selection; QTLs, quantitative trait loci; RGA, rapid generation advancement; SSD, single-seed descent.

## 7 Soybean speed breeding in a cross-crop context

Speed breeding was first developed in long-day and day-neutral crops, with a 22-h photoperiod, early seed harvest and single-seed descent (Ghosh et al., 2018; Watson et al., 2018). This section compares soybean protocols with those of other crops in generation turnover (Section 7.1) and photoperiod requirements (Section 7.2), and identifies which components can be transferred to soybean (Section 7.3).

### 7.1 Generation turnover across crop species

Protocols for several crops give about four to six generations per year (**Table 2**). Cereal protocols reach up to six generations per year, compared with two to three in a conventional glasshouse (Ghosh et al., 2018; Watson et al., 2018). A modified wheat system reaches four while using 80% less energy to maintain the 22-h photoperiod than the existing system (Cha et al., 2023), and lighting recipes for spring wheat grown indoors could potentially give more than seven (Li et al., 2025). In barley, the fast-cycling Golden Promise-rapid line completes a generation in 63 days, 25% shorter than the 84 days of Golden Promise (Buchmann et al., 2026). In soybean, the post-flowering 22-h protocol is reported to give six generations per year, compared with one to three by standard approaches (Aciksoz et al., 2025), and a vegetable-soybean protocol with cycles of 81–92 days was calculated to allow four to four and a half, compared with a conventional one to two (Taku et al., 2024). The short-day requirement therefore does not appear to impose a lower annual ceiling than in long-day cereals. This conclusion, however, rests on comparisons across studies that differ in genotype, facility, harvest timing and seed treatment. Within-study comparisons are limited to the germination of early-harvested seed. Extending the photoperiod after flowering increased germination at 58 days after sowing from 44% under 12 h to 92% under 22 h in an unnamed cultivar (Aciksoz et al., 2025), but by only about 8% at 63 days when the day was extended from 10 to 18 h from day 35, with large differences among genotypes (Jähne et al., 2020). The two studies differ in genotype, photoperiod, timing of the switch and seed treatment, and neither compared cycle length between the extended and short-day treatments. The contribution of the post-flowering long day to the seed-to-seed cycle, as distinct from early harvest and seed treatment, has therefore not been measured directly.

**Table 2:** Speed-breeding and rapid-generation-advancement outcomes in crops other than soybean (see **Table 1** for soybean).

Reference	Crop	Photoperiod response	Protocol	Cycle length	Generations per year
(Ghosh et al., 2018; Watson et al., 2018)	Spring wheat, durum wheat, barley, chickpea, pea, canola	Long-day or day-neutral	22-h photoperiod in a glasshouse or growth chamber; early harvest of immature seed; single-seed descent	—	Up to 6, canola 4 (calculated); 2–3 under normal glasshouse conditions
(Cha et al., 2023)	Spring and winter wheat	Long-day	Speed vernalization, then a 22-h photoperiod in a glasshouse (10 h of sunlight and 12 h of LED light)	—	4 (achieved); 1 in the field
(Li et al., 2025)	Spring wheat	Long-day	Indoor cultivation under white LEDs with a 22-h photoperiod; seed harvested at the milky stage; more than 7 generations per year projected	< 50 d (projected)	Not measured
(Buchmann et al., 2026)	Barley	Long-day	22-h photoperiod; fast-cycling line Golden Promise-rapid; immature spikes harvested	63 d (84 d in Golden Promise)	Up to 6 (calculated)
(Nandigam et al., 2025)	Chickpea	Long-day	12 h of artificial light at night; immature pods harvested 50 days after sowing; single-seed descent	F2 to F7 in 18 months	4 (achieved)
(Gurumurthy et al., 2024)	Chickpea	Long-day	Open field under natural conditions; fresh immature green seed sown	F2 to F5 in 1.5 years	2 per cool season (achieved); 1 conventionally
(Kabade et al., 2024)	Rice	Short-day	24-h photoperiod for the first 15 days, then 10 h; 32/30°C; prematurely harvested seed treated with gibberellic acid	58–71 d	5.1–6.3 (calculated); 1–2 in the field
(Gangashetty et al., 2024)	Pigeonpea	Short-day	13-h, 8-h and 13-h photoperiods at the vegetative, flowering and pod-filling stages; far-red light to trigger flowering; single-seed descent	22–60 d shorter than the control	2–4 (calculated); 1 conventionally
(Sajja et al., 2025)	Finger millet	Short-day	9-h photoperiod; 29°C; 105 plants per 1.5 sq. ft.; harvest at physiological maturity; single-seed descent	64–68 d	4–5 (calculated); 1–2 in the field

Generations per year are marked as calculated or achieved as in **Table 1**, followed by the conventional number where the source gives one. A dash means that the source gives no cycle length.

### 7.2 Photoperiod requirements across crop species

In long-day and day-neutral crops, one extended photoperiod can be kept throughout most of the life cycle. The original protocols use a 22-h light period and a 2-h dark period (Watson et al., 2018), and related conditions have been described for bread and durum wheat, barley, oat, *Brassica* species, chickpea, pea, grass pea and quinoa (Ghosh et al., 2018). The same regime cannot be applied to photoperiod-sensitive soybean. In two cultivars of approximately MG III–IV, a constant 18-h photoperiod delayed flowering to about 63–70 days, and under a constant 22-h photoperiod plants flowered at about 28 days but none reached R6 (Karaman et al., 2026). In soybean the photoperiod therefore has to be set by developmental stage and genotype (Sections 2 and 3.1).

Other short-day crops also need their own photoperiod regimes. Rice is given a 24-h photoperiod for the first 15 days of vegetative growth, followed by a 10-h short day (Kabade et al., 2024). Pigeonpea is given 13-h, 8-h and 13-h photoperiods at the vegetative, flowering and pod-filling stages (Gangashetty et al., 2024), and finger millet a 9-h photoperiod (Sajja et al., 2025).

### 7.3 Transferable and crop-specific components of speed breeding

Some components of speed breeding can be transferred between crops with little modification, whereas others are crop-specific. Early seed harvest and single-seed descent are also used in short-day crops. In rice, the use of prematurely harvested seed and gibberellic acid treatment reduced the maturity duration by 50% (Kabade et al., 2024). In pigeonpea, speed breeding was coupled with single-seed descent and with genotyping of seed or pod chips for marker-assisted selection in early generations (Gangashetty et al., 2024), and the finger millet protocol combines high-density planting with single-seed descent (Sajja et al., 2025). The same components are used in soybean, but the photoperiod regime has to be adapted for each crop.

When protocols are compared, reported generations per year should be considered alongside population size, genotype range, seed viability and the number of cycles validated. Controlled environments are also not the only route to faster generation turnover. In chickpea, a natural speed-breeding approach advanced material from F2 to F5 in 1.5 years in the open field, and can save three years of the conventional six-year timeline for line development (Gurumurthy et al., 2024).

## 8 Conclusions and future perspective

Several RGA protocols are available for soybean (**Table 1**). Up to six generations per year have been reported, as for the original cereal protocols, while four to five are possible without extended photoperiods (**Table 1** and **2**). Under long days, the *E1* family represses *GmFT2a* and *GmFT5a*. Thus, the extended photoperiod used to accelerate wheat development cannot be applied throughout the soybean life cycle. Soybean protocols must account for photoperiod sensitivity. Protocol choice also depends on available infrastructure. Growth chambers provide stage-specific photoperiod control and support integration with marker-assisted and genomic selection. Programs without controlled facilities can use off-site nurseries and fresh seeding, which enabled at least four generations per year (Fang et al., 2021).

The most readily testable question is whether long days after flowering shorten the seed-to-seed cycle, and by how much. Cycle length, germination of early-harvested seeds and seed number per plant should be compared between a post-R1 long-day treatment and a corresponding short-day control, using the same genotypes and facility, preferably across maturity groups. Published controlled-environment soybean RGA protocols do not report changes in allele frequency during accelerated advancement or genetic correlations between traits measured in the chamber and in the field. Genotyping a population before and after advancement and phenotyping it in both environments would help determine whether advancement favors particular genotypes and whether chamber-based selection predicts field performance. Guidelines for controlled-environment drought experiments recommend adequate biological replication, repeated experiments and microclimate measurements (Moshelion et al., 2024). RGA studies should meet the same standards and report cultivar names and maturity groups. Several soybean protocols do not report the maturity group of the plants used (**Table 1**). Ultimately, RGA should be evaluated by realized genetic gain per unit time in the target environment.
